# Low Effectiveness of Prosulfocarb and Mesosulfuron-Methyl + Iodosulfuron-Methyl against *Vulpia myuros*

**DOI:** 10.3390/plants10061186

**Published:** 2021-06-10

**Authors:** Muhammad Javaid Akhter, Abdullatief M. Abdurruhman, Solvejg Kopp Mathiassen, Per Kudsk

**Affiliations:** 1 Research Centre Flakkebjerg, Department of Agroecology, Aarhus University, DK-4200 Slagelse, Denmark; javedakhter8864@gmail.com (M.J.A.); sma@agro.au.dk (S.K.M.); 2 Department of Crop Protection, Faculty of Agriculture, University of Khartoum, Khartoum 11115, Sudan; abdullatief1213@gmail.com; 3 Centre of Excellence Pesticides and Plant Health (PPH), Univerity of khartoum, Khartoum 11115, Sudan

**Keywords:** natural tolerance, chemical control, integrated weed management

## Abstract

Due to natural tolerance to most widely used herbicides for grass weed control, prosulfocarb as pre-emergence or early post-emergence herbicide and mesosulfuron + iodosulfuron as post-emergence herbicide are the mainstays of any chemical control program for *Vulpia myuros* in Denmark. However, farmers often report variable efficacy of these herbicides on *V. myuros* compared to other grass weeds. Dose–response experiments were conducted to evaluate the performance of prosulfocarb and mesosulfuron + iodosulfuron on *V. myuros.* Prosulfocarb was sprayed at different plant growth stages to study the influence of plant growth stage on the performance of prosulfocarb on *V. myuros* in comparison with the more susceptible grass weed species *Apera spica-venti*. Doses causing 50% reduction in response variable (ED_50_) were estimated from the dose–response analysis. The ED_50_ values revealed a higher tolerance of *V. myuros* to prosulfocarb and mesosulfuron + iodosulfuron than *A. spica-venti.* The relative difference in the effectiveness of prosulfocarb between *V. myuros* and *A. spica-venti* was constant among plant growth stages studied. The highest levels of *V. myuros* control were achieved when prosulfocarb was sprayed pre-emergence (BBCH 00), while the control substantially declined at later growth stages. The results from the current study document the tolerance of *V. myuros* to prosulfocarb and mesosulfuron + iodosulfuron and highlight the importance of optimization of prosulfocarb spray timing for achieving maximum control of *V. myuros*.

## 1. Introduction

Rattail fescue (*Vulpia myuros* (L.) C.C. Gmel.) is considered a problematic grass weed in parts of Northern Europe including Denmark, where its recent increase in abundance is due to a high frequency of winter cereals in crop rotations and wide adoption of no tillage practices [1]. Recently, *V. myuros* has been reported at densities of several hundred plants/m^2^ in winter cereals in Denmark [2]. *V. myuros* also has been reported in other EU countries, for example, in United Kingdom, France, Germany, Romania, and Switzerland, however it is still considered a minor weed in those countries [3].

*V. myuros,* a self-pollinating winter annual grass weed species, has a life cycle very similar to winter wheat [4]. It has a high fecundity and can germinate quickly and under a wide range of conditions [5]. Competition of *V. myuros* can cause significant grain yield losses in a range of crops [6]. A study conducted in Denmark showed that *V. myuros* at 405 plants/m^2^ can cause yield losses up to 50% in winter wheat [7].

*V. myuros* traditionally has been managed by plowing in conventional farming systems. However, the adoption of no tillage cropping systems to preserve soil productivity and reduce energy use eliminates the possibility of using tillage to control *V. myuros*. Acetyl-coenzyme A carboxylase (ACCase) and acetolactate synthase (ALS) inhibitors are the most widely used herbicides to control grass weeds in winter cereals. However, *V. myuros* is naturally tolerant to ACCase inhibitors, and many ALS inhibitor herbicides are not highly effective against this weed species [2,8,9]. Previously, several studies have related the poor performance of foliar applied herbicides on *V. myuros* to low spay retention on the narrow leaf blades of this species [10,11].

Due to natural tolerance to most widely used herbicides, the options for chemical control of *V. myuros* are limited compared with other grass weeds. In Denmark, farmers rely on residual herbicides such as prosulfocarb applied pre-emergence or early post-emergence and post-emergence application of mesosulfuron + iodosulfuron in autumn and/or spring for the control of *V. myuros* in winter cereals. However, some reports suggest that the typically used rates of prosulfocarb and mesosulfuron + iodosulfuron provide more inconsistent control of *V. myuros* than of other grasses [2,8,12]. The effectiveness of control with prosulfocarb depends on the growth stage of *V. myuros*. For example, previous studies have shown that some level of *V. myuros* control could be achieved if prosulfocarb was applied pre-emergence, but effectiveness tended to decrease rapidly when sprayed at later growth stages [8,13]. However, the information on prosulfocarb performance as an early post-emergence application on *V. myuros* is limited. To optimize herbicide application timings, information on prosulfocarb effectiveness on *V. myuros* at different growth stages is required. Reports suggest that *V. myuros* can tolerate the recommended rates of mesosulfuron + iodosulfuron better than other grass weed species such as *A. spica-venti* [2], but there is no information on the level of tolerance in *V. myuros* against mesosulfuron + iodosulfuron. The objective of this study was to assess the performance of prosulfocarb and mesosulfuron + iodosulfuron and evaluate the impact of plant growth stages on the performance of prosulfocarb on *V. myuros*. *Apera spica-venti* was used as a susceptible control species in the study.

## 2. Materials and Methods

### 2.1. Seed Source

Seeds of *V. myuros* were collected from a non-agricultural area at Flakkebjerg Research Centre, Denmark, where there has been no known history of herbicide application. Seeds of susceptible populations of *A. spica-venti* originated from several locations in Denmark and were mixed together in the same proportion to create a meta-population [14].

### 2.2. Prosulfocarb Bioassay

Twenty seeds of *V. myuros* and *A. spica-venti* were sown in 1 L pots filled with field soil (sandy loam). The pots were placed in an unheated glasshouse or on outdoor tables depending on the time of the year when the experiment was conducted. Groups of pots were sown on different dates to ensure different plant growth stages could be sprayed at the same time. After seedling emergence, the number of plants per pot were thinned to a pre-set number. Pots treated pre-emergence were not thinned. Pre-emergence treatments were carried out one day after sowing. Plants were treated with a range of prosulfocarb doses (0, 25, 50, 100, 200, 400, 800, 1000, 2000, and 4000 g/ha) (Boxer, 800 g L^−1^ prosulfocarb, Syngenta Crop Protection A/S, Denmark). Herbicide application was carried out in a spray cabinet equipped with a boom fitted with two flat-fan nozzle (HARDI ISO F-110-02). The nozzles were operated at a pressure of 300 kilopascal and velocity of a 5.2 km h^−1^ delivering spray volume of a 152 L ha^−1^. A few hours after application of prosulfocarb, 30 mL of Milli Q water was applied to the soil surface of each pot to ensure a uniform distribution of prosulfocarb in the upper soil layer. The plants were sprayed at BBCH (Biologische Bundesanstalt Bundessortenamt and Chemical Industry) 00 to 13 (Table 1). The prosulfocarb dose–response study was repeated four times. As plants were sprayed at different growth stages in four repeats, thus making the interpretation of results straightforward. Experiments 1–4 hereafter refer to the four runs of the dose–response study (Table 1). The four experiments were sprayed on 22 May 2018 (Experiment 1), 23 October 2018 (Experiment 2), 18 March 2019 (Experiment 3), and 15 April 2019 (Experiment 4). After spraying, the pots were placed in growth chamber at 14/10 °C day/night with 16 h photoperiods for Experiment 1, in a cold glasshouse for Experiments 2 and 3, and outdoors for Experiment 4 (Table 1). There were three replicates per treatment plus an untreated control in a complete randomized block design. Dry foliage weights were recorded 3–4 weeks after herbicide treatment.

### 2.3. Mesosulfuron-Methyl + Iodosulfuron-Methyl Bioassay

#### 2.3.1. Seed Germination Assay

The plant agar medium (Duchefa) (10 g/L) (1% *w*/*v*) was boiled in a microwave and allowed to cool to 35–40 °C. A stock solution of mesosulfuron-methyl + iodosulfuron-methyl (Atlantis OD, 10 g/L mesosulfuron-methyl + 2 g/L iodosulfuron-methyl + 30 g/L mefenpyr-diethyl, Bayer CropScience, Denmark) in the commercial form was prepared. Using this stock solution, different concentrations of mesosulfuron-methyl + iodosulfuron-methyl were prepared. Mesosulfuron-methyl + iodosulfuron-methyl assay concentrations were 0, 0.009, 0.018, 0.036, 0.072, and 0.144 mg/L. The herbicide concentrations were added to agar medium using micropipette. Twenty milliliters of agar medium plus herbicide solution were poured into quadratic plastic dishes (245 mm × 245 mm). Fifty seeds of *V. myuros* and *A. spica-venti* were placed in each dish. The dishes were covered with lids and placed in the greenhouse. The experiment was arranged in a completely randomized design with three replicates. Coleoptile and radicle lengths were measured 10–15 days after treatment.

#### 2.3.2. Seedling Assay 

Plants of *V. myuros* and *A. spica-venti* were grown in plastic trays using potting mixture containing soil, peat, and sand (2:1:1 by weight). The plastic trays were placed in an unheated glasshouse on the table with automatic watering system. The plant agar media and herbicide concentrations were prepared as described above in the seed germination assay. Seedlings of *V. myuros* and *A. spica-venti* at the 2–3 leaf stage (BBCH 12–13) were harvested from the plastic trays and washed under tap water to remove any particles of potting mixture from roots. The seedling tips (growing points of leaves and roots) were excised. Roots of seedlings were transplanted into plastic dishes (245 mm × 245 mm) containing a plant agar media using forceps. The plastic dishes were covered and placed in the heated glasshouse. The experiment was arranged in a completely randomized design with ten plant seedlings in each dish and three replications. Herbicide efficacy was recorded as seedling survival by visually assessing the new roots and shoots 15 days after transplanting.

### 2.4. Statistical Analysis

A nonlinear log-logistic model was used to analyze the prosulfocarb and mesosulfuron + iodosulfuron dose–response studies [15]:

(1) $$Y=\frac{d-c}{1+exp\left[b\left(log\left(t\right)-log\left(e\right)\right)\right]}$$

where *Y* is the response variable (represents the percent fresh weight, germination, coleoptile length, and radicle length relative to untreated control); *c* and *d* denote the lower and upper asymptote, respectively; *e* is the dose causing 50% reduction in response variable (ED_50_); and *b* is the slope of the curve. The model was checked by residual plots and with lack of fit test (*p* > 0.05). If the value of *c* was zero, the four-parametric model was reduced to a three-parametric one as:

(2) $$Y=\frac{d}{1+exp\left[b\left(log\left(t\right)-log\left(e\right)\right)\right]}$$

The analyses were performed using R version 3.6.1 (R Foundation for Statistical Computing, Vienna, Austria, http://R-project.org) with drc package [16]. The value of *e* (ED_50_) was compared between the species using post hoc *t*-tests. Tolerance index (TI) was estimated as *V. myuros* to *A. spica-venti* ED_50_ ratios to compare the responses from a population of *V. myuros* with those of *A. spica-venti*.

## 3. Results

### 3.1. Prosulfocarb Bioassay

**Figure 1:**
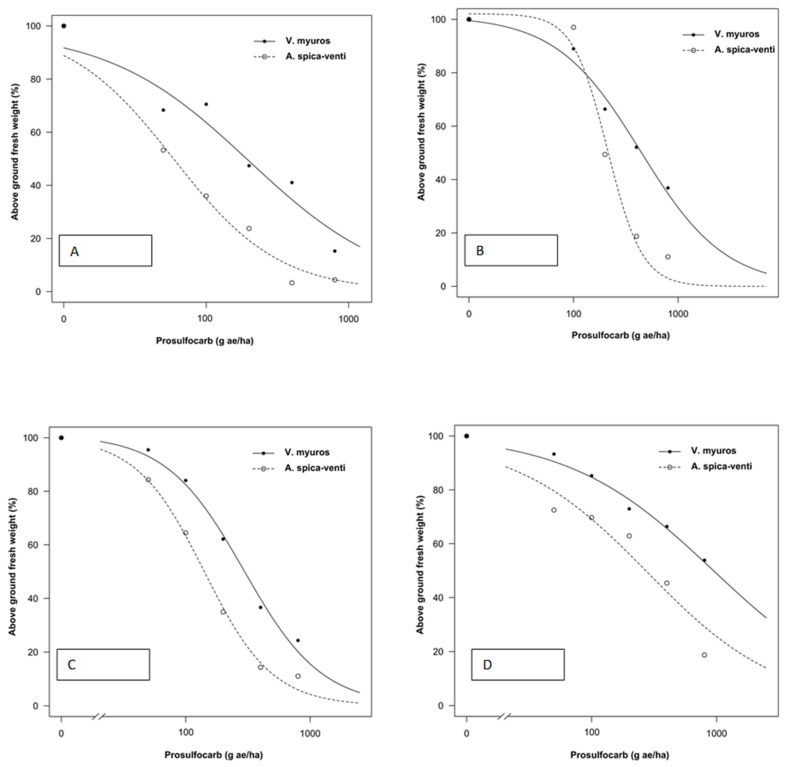
Prosulfocarb dose–response on aboveground fresh weight represented as percentage of untreated control of *Vulpia myuros* and *Apera spica-venti* in Experiment 1 at: BBCH 00 (**A**); BBCH 11 (**B**); BBCH 12 (**C**); and BBCH 13 (**D**).

**Figure 2:**
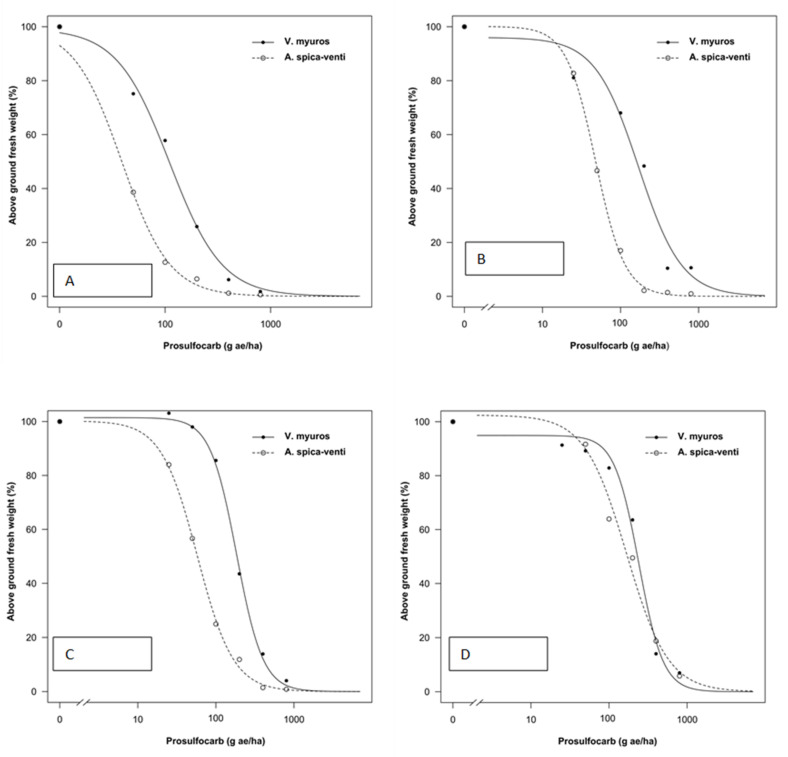
Prosulfocarb dose–response on aboveground fresh weight represented as percentage of untreated control of *Vulpia myuros* and *Apera spica-venti* in Experiment 2 at: BBCH 00 (**A**); BBCH 10 (**B**); BBCH 11 (**C**); and BBCH 12 (**D**).

**Figure 3:**
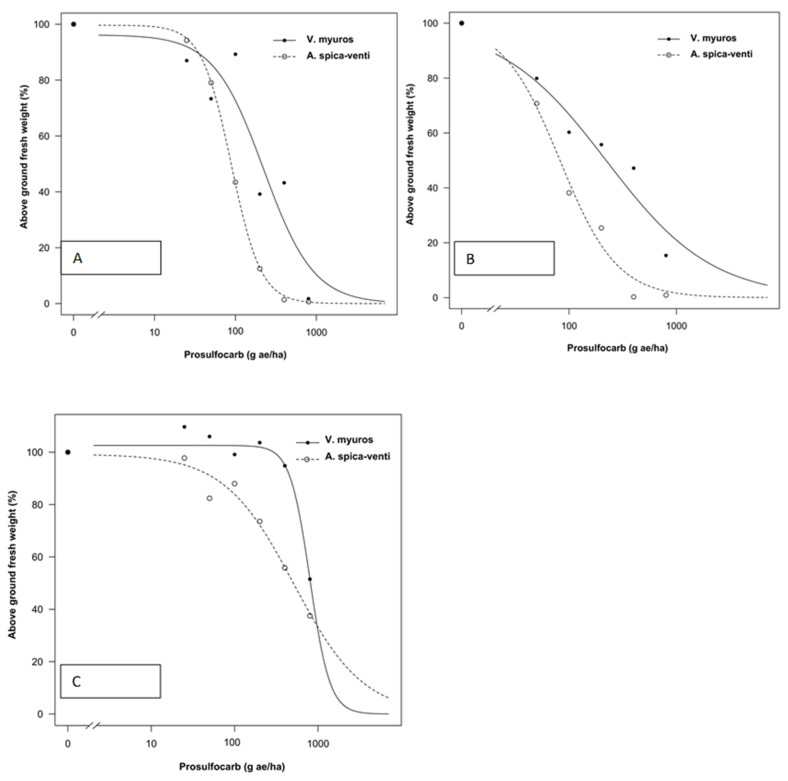
Prosulfocarb dose–response on aboveground fresh weight represented as percentage of untreated control of *Vulpia myuros* and *Apera spica-venti* in Experiment 3 at: BBCH 00 (**A**); BBCH 10 (**B**); and BBCH 13 (**C**).

**Figure 4:**
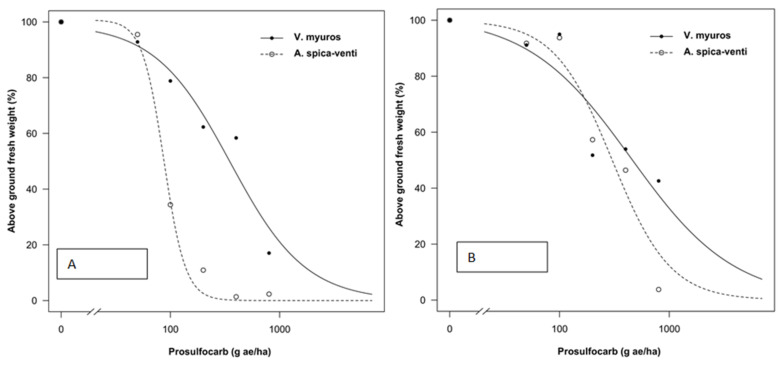
Prosulfocarb dose–response on aboveground fresh weight represented as percentage of untreated control of *Vulpia myuros* and *Apera spica-venti* Experiment 4 at: BBCH 11 (**A**); and BBCH 12 (**B**).

**Figure 5:**
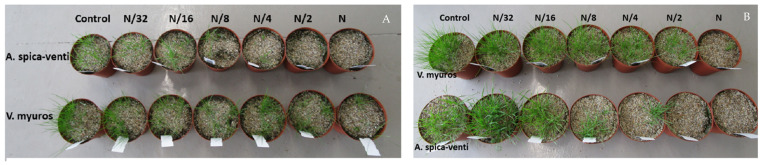
Photographic illustration of prosulfocarb dose–response for the *Vulpia myuros* and *Apera spica-venti* sprayed on: pre-emergent seeds (**A**); and germinating seedlings (**B**). The normal dose (N) of prosulfocarb was 800 g/ha.

Data from all experiments were analyzed separately as plants were sprayed at different growth stages and significant differences were found in the regression parameter estimates between experiments. The tolerance index (TI), i.e., ED_50_ value of *V. myuros* relative to ED_50_ value of *A. spica-venti*, expressing the level of herbicide tolerance in *V. myuros* relative to *A. spica-venti*, was calculated by dividing the estimated ED_50_ values for *V. myuros* by the corresponding values for *A. spica-venti* (Figure 1, Figure 2, Figure 3 and Figure 4). TI’s values showed that *V. myuros* was more tolerant to prosulfocarb than *A. spica-venti* at all growth stages studied (Table 2 and Figure 5). *V. myuros* showed 3.4-, 2.1-, 2.1-, and 3.3-fold tolerance at the BBCH 00 (pre-emergence), BBCH 11, BBCH 12, and BBCH 13 stages, respectively, in Experiment 1. *V. myuros* exhibited 2.8-, 3.5-, 3.2-, and 1.4-fold tolerance at the BBCH 00, BBCH 10, BBCH 11, and BBCH 12 stages, respectively, in Experiment 2 (Table 2). The TI for *V. myuros* was 2.5-, 2.7-, and 1.6-fold at the BBCH 00, BBCH 10, and BBCH 13 stages, respectively, in Experiment 3 (Table 2). In Experiment 4, *V. myuros* showed 4.0- and 1.5-fold tolerance at the BBCH 11 and BBCH 13 stages, respectively.

Application rates of prosulfocarb required to suppress 50% shoot biomass of *V. myuros* was lowest at BBCH 00 stage and, with a few exceptions, tended to increase at more advanced growth stages. Generally, a similar trend was observed for *A. spica-venti*. For instance, the estimated ED_50_ value was lower at the BBCH 00 stage than at BBCH 11, BBCH 10, and BBCH 13 in Experiments 1–3, respectively. In Experiment 4, the plants were sprayed only at two growth stages (BBCH 11 and BBCH 13), where the rates of prosulfocarb for 50% shoot biomass reduction of *V. myuros* was lower at BBCH 11 than at BBCH 13, although the difference was non-significant. For *A. spica-venti,* the estimated ED_50_ values were significantly lower at BBCH 11 than at BBCH 13 in Experiment 4. In general, moving towards more advanced growth stages, higher ED_50_ values were estimated than at early growth stages, and these results are consistent in the two species and four trials.

### 3.2. Mesosulfuron-Methyl + Iodosulfuron-Methyl Bioassay

#### 3.2.1. Seed Germination Assay in Agar Medium

**Figure 6:**
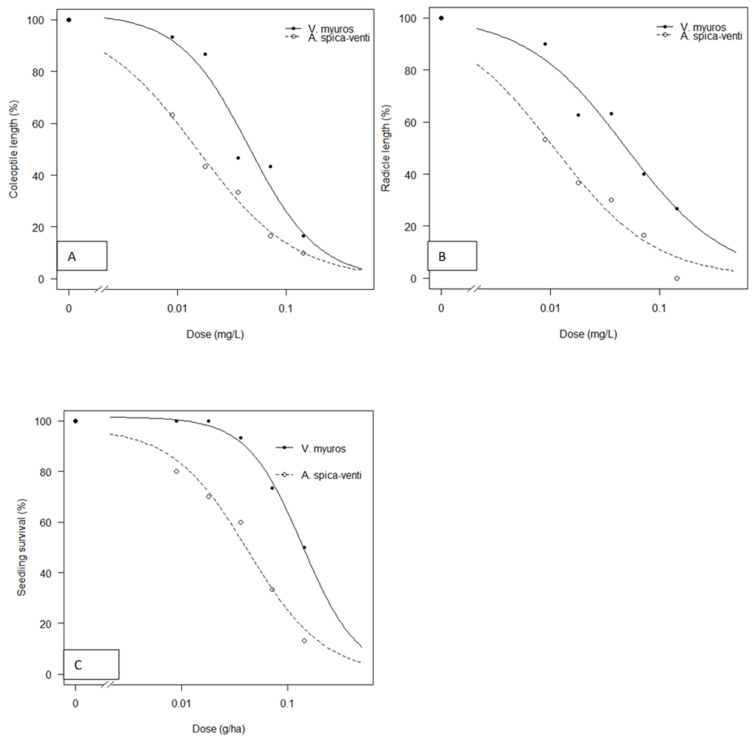
Dose–response of mesosulfuron-methyl + iodosulfuron-methyl on germination and coleoptile (**A**) and radicle lengths (**B**) and seedling survival (**C**) of *V. myuros* and *A. spica-venti* in agar-based bioassay.

Coleoptile and radicle lengths were inhibited by herbicide treatments for both grass weed species, but the inhibition was significantly higher for *A. spica-venti* than for *V. myuros* (Figure 6A,B). Estimated ED_50_ values for *V. myuros* and *A. spica-venti* were 0.04 and 0.01 mg/L for coleoptile growth and 0.05 and 0.01 mg/L for radicle growth, respectively (Table 3). The ED_50_ of *V. myuros* was 3.0 and 4.4 times (TI) higher than *A. spica-venti* for coleoptile and radicle growth, respectively.

#### 3.2.2. Seedling Assay

**Figure 7:**
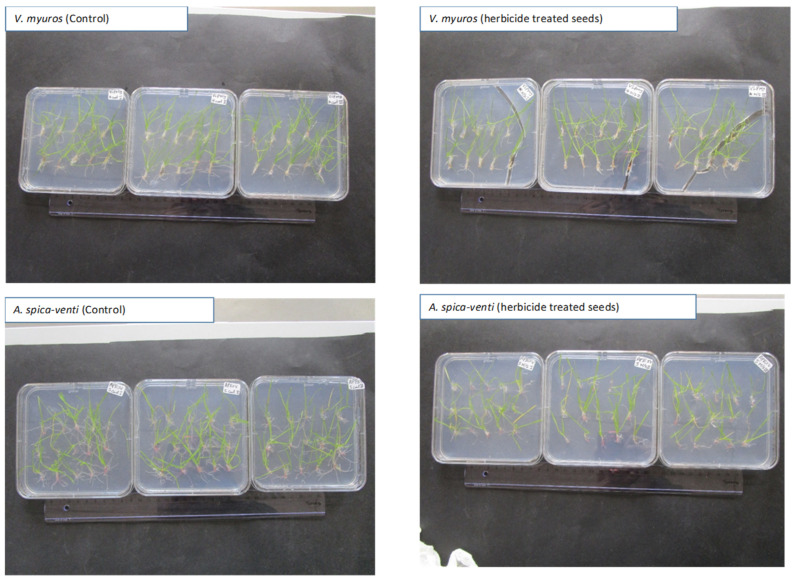
Photographic illustration of *V. myuros* and *A. spica-venti* populations assayed as untreated control and at 0.072 mg/L mesosulfuron + iodosulfuron in the agar-based seedling assay.

The plant agar was found to be a suitable media for growing *V. myuros* and *A. spica-venti*, where individuals from both *V. myuros* and *A. spica-venti* developed new roots and shoots. New roots and shoots of both species were suppressed more in dishes with herbicide compared to untreated controls, but the suppression of new roots and shoots was significantly higher in *A. spica-venti* than in *V. myuros* (Figure 6C and Figure 7). For instance, the ED_50_ values for *V. myuros* and *A. spica-venti* were 0.14 and 0.04 mg/L, respectively, indicating that *V. myuros* was 3.2 times (TI) more tolerant to mesosulfuron + iodosulfuron than *A. spica-venti* (Table 3). The results from both seed germination and seedling assay show that *V. myuros* is more tolerant to mesosulfuron + iodosulfuron than *A. spica-venti*.

## 4. Discussion

Due to natural tolerance to most of the widely used herbicides, prosulfocarb and mesosulfuron + iodosulfuron are considered important chemical control options for *V. myuros*. Nonetheless, farmers have reported that the two herbicides are not highly effective in controlling *V. myuros* compared with other grass weeds such as *A. spica-venti*. To evaluate the level of tolerance of *V. myuros* to prosulfocarb and mesosulfuron + iodosulfuron, we carried out outdoor, glasshouse, and laboratory studies on a *V. myuros* population and compared the responses to a meta-population of *A. spica-venti*.

### 4.1. Prosulfocarb Bioassay

**Figure 8:**
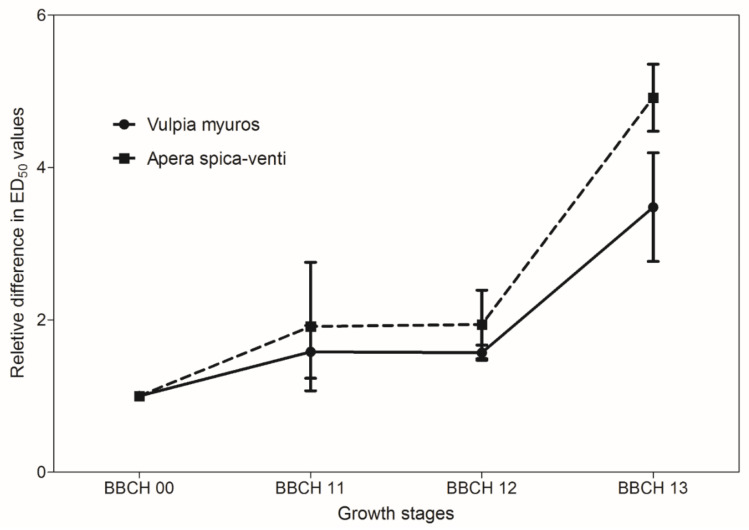
The relationship between relative difference in ED_50_ values with the increased growth stages and BBCH growth stages in *V. myuros* and *A. spica-venti*. The ED_50_ values with the increased growth stages were pooled across Experiments 1–3. Vertical bars represent standard errors of the mean.

The results obtained in the prosulfocarb dose–response studies showed a higher tolerance of *V. myuros* to prosulfocarb compared with *A. spica-venti* (Table 2 and Figure 1, Figure 2, Figure 3 and Figure 4). Other researchers also found that prosulfocarb is not highly effective in controlling *V. myuros*, and often maximum authorized doses were needed for achieving some level of control [8,17]. The results of the three experiments are not comparable because the experiments were carried out at different times of the year and, thus, under different climatic conditions. The primary aim of this study was to evaluate the level of susceptibility to prosulfocarb of different growth stages of *V. myuros* to that of *A. spica-venti* at the same growth stages. The results are consistent across experiments with *V. myuros* being less susceptible than *A. spica-venti.* To visualize the relative increase in prosulfocarb dose required to suppress 50% biomass at later growth stages in *V. myuros* and *A. spica-venti,* the estimated relative ED_50_ values with the increased growth stages were pooled across experiments and plotted against BBCH growth stage for the two weed species (Figure 8). The ED_50_ values from Experiment 4 are not included in the plot because in Experiment 4 plants were not sprayed at the pre-emergence stage, and, therefore, it was not possible to calculate the relative ED_50_ values. The plot between relative ED_50_ values and BBCH growth stages showed that relative difference in the effectiveness of prosulfocarb between *V. myuros* and *A. spica-venti* tends to remain similar when sprayed at different plant growth stages, which indirectly indicate that tolerance in *V. myuros* is inherent and tends to remain constant at different growth stages. More research is needed to investigate the mechanism of tolerance in *V. myuros* in detail and should include absorption, translocation, metabolism, and vacuolar sequestration studies.

The results show that weed growth stage at the time of spraying had a significant effect on plant sensitivity to prosulfocarb. Pre-emergence application of prosulfocarb at the rate of 800 g/ha resulted, on average, in an 80–90% biomass reduction of *V. myuros*, while the same herbicide dose provided 70–80% dry matter reduction when sprayed at emerged seedling stages (BBCH 10 to BBCH 11). Similar to our results, Hull et al., [8] also found a lower efficacy of prosulfocarb when applied early post-emergence compared to pre-emergence applications.

In the current study, the estimated ED_50_ doses required to control *V. myuros* and *A. spica-venti* were lower than the typically used field doses. Pot-grown weed plants are generally more susceptible to herbicides than plants grown in the field and, interestingly, results from the four experiments are consistent, indicating lower susceptibility of *V. myuros* to prosulfocarb, regardless of plant growth stage. Farmers often experience failures when applying prosulfocarb for the control of *V. myuros* under field conditions, and field observations have shown that significantly higher doses of prosulfocarb are needed for effective control. In contrast, prosulfocarb provided effective control of *A. spica-venti* with ED_50_ values of less than 100 g ha^−1^ at pre-emergence (BBCH 00) and BBCH 10 and less than 200 g ha^−1^ at BBCH 12. However, beyond BBCH 12, control of *A. spica-venti* with prosulfocarb tended to decline. Our findings are in line with those of Adamczewski et al. [18] who also reported satisfactory control of *A. spica-venti* with recommended rates of prosulfocarb when applied pre-emergence under field conditions.

### 4.2. Mesosulfuron-Methyl + Iodosulfuron-Methyl Bioassay

The agar-based assay showed that the lengths of coleoptiles and radicles and seedling survival of *V. myuros* and *A. spica-venti* were suppressed by increasing doses of mesosulfuron-methyl + iodosulfuron-methyl. Abdurruhman et al. [19] reported suppression of coleoptile and radicle lengths and establishment of new shoots and roots in wild oat (*Avena sterilis* L.) populations treated with mesosulfuron-methyl + iodosulfuron-methyl in agar media. Previously, many studies have shown that the agar-based assay can be used to detect resistance in grass weeds such as *Sorghum halepense*, *Alopecurus myosuroides,* and *Lolium rigidum* [20,21,22]. This method has proven to be a reliable and quick way to detect the differences between resistant/tolerant and susceptible grass weed biotypes for pre-emergence and post-emergence herbicides [19,23]. The results obtained in agar-based assay show that the ED_50_ values for *V. myuros* were 3–4.4 times higher than for *A. spica-venti*. The results confirm that *V. myuros* is tolerant to mesosulfuron-methyl + iodosulfuron-methyl, as it is to other herbicides. Other researchers found that mesosulfuron-methyl + iodosulfuron-methyl is not highly effective in controlling *V. myuros*, and often higher than the maximum recommended rates were needed for satisfactory control of this weed species [8]. It is often anticipated that ineffective control of *V. myuros* with post-emergence herbicides can be attributed to low spray retention due to its narrow and erect leaves [10,11]. The results of the agar-based test show that *V. myuros* is naturally tolerant to mesosulfuron-methyl + iodosulfuron-methyl and that differences in spray deposition can only partly be the cause for the observed differences in susceptibility. This assumption is further supported by the finding of Akhter, [24], who showed that low spray retention is not the cause of low performance of glyphosate on *V. myuros*. Yu et al. [9], studying the closely related species *V. bromoides*, proved its tolerance to ACCase and several ALS inhibitors and concluded that an insensitive ACCase and enhanced metabolism were the likely mechanisms of tolerance to the two modes of action. Here, we document for first the time that *V. myuros*, similar to *V. bromoides*, is naturally tolerant to mesosulfuron-methyl + iodosulfuron-methyl compared to a population of *A. spica-venti*. In the current study, we did not attempt to study the *V. myuros* tolerance mechanism against mesosulfuron-methyl + iodosulfuron-methyl, but the available information shows that the response of *V. myuros* and *V. bromoides* to various herbicides are similar. Hence, it can be speculated that the mechanism of tolerance in *V. myuros* to ALS inhibiting herbicides including mesosulfuron-methyl + iodosulfuron-methyl might be due the same mechanism as found for *V. bromoides* [2].

According to the current study, pre-emergence application of prosulfocarb can provide some level of *V. myuros* control but prosulfocarb has a narrow window of application as the activity declined significantly at more advanced growth stages. Moreover, the soil half-life of some residual herbicides is short, for example, the half-life of prosulfocarb is around 10 days [25], and, therefore, pre-emergence herbicide spraying potentially cannot control late germinating *V. myuros* plants, particularly in years with mild winters and high dormancy. Changing from a single application to sequential applications of herbicides can overcome this shortcoming and provide more effective control of other grass weeds. Nonetheless, the results from the current study indirectly indicate that *V. myuros* control with sequential treatments of herbicides, i.e., combining a pre-emergence application of prosulfocarb with a post-emergence treatment of mesosulfuron-methyl + iodosulfuron-methyl, would be less than acceptable, especially on dense stands. In the absence of effective chemical control methods, *V. myuros*, due to its profound growth behavior, can quickly take over the field and cause significant grain yield losses [7]. It appears that management of *V. myuros* will require other inputs in addition to herbicides. Integrated weed management strategies with several preventive and cultural control measures such as tillage, crop rotation, delayed crop sowing, and crop competition applied in combination with available herbicides can facilitate sustainable weed management of *V. myuros*.

## 5. Conclusions

The current study revealed that *V. myuros* is tolerant to prosulfocarb and mesosulfuron-methyl + iodosulfuron-methyl compared with *A. spica-venti,* and the relative differences in the effectiveness of prosulfocarb between *V. myuros* and *A. spica-venti* tend to remain constant among the plant growth stages studied. The results further show that the highest level of *V. myuros* control with prosulfocarb is achieved if the herbicide is applied pre-emergence or on germinating seedlings, whereas control tends to decline substantially beyond these growth stages. The information on the level of *V. myuros* tolerance to herbicides documented here will help for planning a proper chemical weed control program as part of integrated weed management strategies against this species.

## Figures and Tables

**Table 1:** A table displaying the set-up of different experiments carried out to examine the level of the lower performance of prosulfocarb and mesosulfuron-methyl + iodosulfuron-methyl in *V. myuros*.

Study	Experiment	Treatments (Herbicide Dose and/or Plant Stages)	Environment
Prosulfocarb bioassay	Experiment 1	Prosulfocarb rate ranging from 0 to 4000 g ha^−1^; BBCH 00, BBCH 11, BBCH 12, BBCH 13	Growth chamber at 14/10 °C day/night with 16 h photoperiods
	Experiment 2	Prosulfocarb rate ranging from 0 to 4000 g ha^−1^; BBCH 00, BBCH 10, BBCH 11, BBCH 12	Unheated glasshouse
	Experiment 3	Prosulfocarb rate ranging from 0 to 4000 g ha^−1^; BBCH 00, BBCH 10, BBCH 13	Unheated glasshouse
	Experiment 4	Prosulfocarb rate ranging from 0 to 4000 g ha^−1^; BBCH 11, BBCH 12	Outdoor under natural conditions
Mesosulfuron-methyl + iodosulfuron-methyl bioassay	Seed germination assay	Mesosulfuron-methyl + iodosulfuron-methyl concentrations ranging from 0 to144 mg/L were added to agar medium containing weed seeds	Laboratory
	Seedling assay	Mesosulfuron-methyl + iodosulfuron-methyl concentrations ranging from 0 to144 mg/L were added to agar medium containing weed seedlings	Laboratory

**Table 2:** Prosulfocarb doses (g ha^−1^) providing 50% reduction in fresh weight of *V. myuros* and *A. spica-venti* estimated using log-logistic model. Standard errors are presented in parentheses.

Growth Stage		BBCH 00 (1)	BBCH 11 (2)	BBCH 12 (3)	BBCH 13 (4)	Significance Level between Growth Stages
**Experiment 1**	*V. myuros*	199 (43.9)	438 (87.6)	293 (26.6)	926 (252.2)	1 vs. 2, *p* < 0.001;1 vs. 3, *p* = 0.074; 1 vs. 4, *p* < 0.001; 2 vs. 3, *p* = 0.144; 2 vs. 4, *p* = 0.003; 3 vs. 4, *p* < 0.001;
	*A. spica-venti*	59 (10.4)	212 (19.3)	141 (11.7)	278 (48.3)	1 vs. 2, *p* = 0.04; 1 vs. 3, *p* = 0.002; 1 vs. 4, *p* < 0.001; 2 vs. 3, *p* = 0.006; 2 vs. 4, *p* = 0.086; 3 vs. 4, *p* < 0.001
Tolerance indices	TI	3.4 *p* = 0.017	2.1 *p* = 0.026	2.1 *p* < 0.001	3.3 *p* = 0.038	
**Growth stage**		**BBCH 00**	**BBCH 10**	**BBCH 11**	**BBCH 12**	
**Experiment 2**	*V. myuros*	110.2 (8.9)	170 (18.6)	184 (12.6)	241 (17.0)	1 vs. 2, *p* = 0.17; 1 vs. 3, *p* = 0.004; 1 vs. 4, *p* < 0.001; 2 vs. 3, *p* = 0.686; 2 vs. 4, *p* = 0.041; 3 vs. 4, *p* = 0.038; 3 vs. 4, *p* < 0.001
	*A. spica-venti*	39 (5.3)	48 (4.03)	58 (5.1)	167 (15.6)	1 vs. 2, *p* = 0.17; 1 vs. 3, *p* = 0.004; 1 vs. 4, *p* < 0.001; 2 vs. 3, *p* = 0.076; 2 vs. 4, *p* < 0.001; 3 vs. 4, *p* < 0.001; 3 vs. 4, *p* < 0.001
Tolerance indices	TI	2.8 *p* < 0.001	3.5 *p* < 0.001	3.2 *p* < 0.001	1.4 *p* = 0.012	
**Growth stage**		**BBCH 00**	**BBCH 10**	**BBCH 13**		
**Experiment 3**	*V. myuros*	223 (46.9)	223 (50.9)	802 (54.6)		1 vs. 2, *p* = 0.948; 1 vs. 3, *p* < 0.001; 2 vs. 3, *p* < 0.001
	*A. spica-venti*	89 (12.4)	82 (11.7)	513 (90.8)		1 vs. 2, *p* = 0.669; 1 vs. 3, *p* < 0.001; 2 vs. 3, *p* < 0.001
Tolerance indices	TI	2.5 *p* = 0.021	2.7 *p* = 0.024	1.6 *p* = 0.064		
**Growth stage**		**BBCH 11**	**BBCH 12**			
**Experiment 4**	*V. myuros*	353 (63.5)	447.3 (135.8)			1 vs. 2, *p* = 0.3716
	*A. spica-venti*	87.1 (7.3)	302.4 (58.7)			1 vs. 2, *p* < 0.001
	TI	4.0 *p* < 0.001	1.5 *p* = 0.3751			

**Table 3:** Mesosulfuron-methyl + iodosulfuron-methyl-sodium rates (mg/L) providing 50% reduction of response variable using log-logistic model for *V. myuros* and *A. spica-venti* in agar-based seed germination and seedling assay. Standard errors are presented in parentheses.

Experiments	Species	*V. myuros*	*Apera Spica-Venti*	Tolerance Index
Seed germination assay	Coleoptile length	0.04 (0.007)	0.01 (0.003)	3.0 *p* = 0.021
	Radicle length	0.05 (0.008)	0.01 (0.002)	4.4 *p* = 0.006
Seedling assay	Seedling survival	0.14 (0.023)	0.04 (0.009)	3.2 *p* = 0.017
